# Enhancing ^18^F-FDG PET image quality and lesion diagnostic performance across different body mass index using the deep progressive learning reconstruction algorithm

**DOI:** 10.1186/s40644-025-00877-x

**Published:** 2025-05-01

**Authors:** Zhihao Chen, Hongxing Yang, Ming Qi, Wen Chen, Fei Liu, Shaoli Song, Jianping Zhang

**Affiliations:** 1 Department of Nuclear Medicine, Fudan University Shanghai Cancer Center, Shanghai, 200032 China; 2 Department of Oncology, Shanghai Medical College, Fudan University, Shanghai, 200032 China; 3 Center for Biomedical Imaging, Fudan University, Shanghai, 200032 China; 4 Shanghai Engineering Research Center for Molecular Imaging Probes, Shanghai, 200032 China; 5 Key Laboratory of Nuclear Physics and Ion-beam Application (MOE), Fudan University, Shanghai, 200433 China; 6 Shanghai Key Laboratory of Bioactive Small Molecules, Fudan University, Shanghai, 200032 China

**Keywords:** PET image quality, Deep progressive learning (DPL), Ordered subset expectation maximization (OSEM), BMI, Lesion diagnostic performance

## Abstract

**Background:**

As body mass index (BMI) increases, the quality of 2-deoxy-2-[fluorine-18]fluoro-D-glucose (^18^F-FDG) positron emission tomography (PET) images reconstructed with ordered subset expectation maximization (OSEM) declines, negatively impacting lesion diagnostics. It is crucial to identify methods that ensure consistent diagnostic accuracy and maintain image quality. Deep progressive learning (DPL) algorithm, an Artificial Intelligence(AI)-based PET reconstruction technique, offers a promising solution.

**Methods:**

150 patients underwent ^18^F-FDG PET/CT scans and were categorized by BMI into underweight, normal, and overweight groups. PET images were reconstructed using both OSEM and DPL and their image quality was assessed both visually and quantitatively. Visual assessment employed a 5-point Likert scale to evaluate overall score, image sharpness, image noise, and diagnostic confidence. Quantitative assessment parameters included the background liver image-uniformity-index ($\:{\text{I}\text{U}\text{I}}_{\text{L}\text{i}\text{v}\text{e}\text{r}}$) and signal-to-noise ratio ($\:{\text{S}\text{N}\text{R}}_{\text{L}\text{i}\text{v}\text{e}\text{r}}$). Additionally, 466 identifiable lesions were categorized by size: sub-centimeter and larger. We compared maximum standard uptake value ($\:{\text{S}\text{U}\text{V}}_{\text{m}\text{a}\text{x}}^{\text{L}\text{e}\text{s}\text{i}\text{o}\text{n}}$), signal-to-background ratio ($\:{\text{S}\text{B}\text{R}}_{\text{L}\text{e}\text{s}\text{i}\text{o}\text{n}}$), $\:{\text{S}\text{N}\text{R}}_{\text{L}\text{e}\text{s}\text{i}\text{o}\text{n}}$, contrast-to-background ratio ($\:{\text{C}\text{B}\text{R}}_{\text{L}\text{e}\text{s}\text{i}\text{o}\text{n}}$), and contrast-to-noise ratio ($\:{\text{C}\text{N}\text{R}}_{\text{L}\text{e}\text{s}\text{i}\text{o}\text{n}}$) of these lesions to evaluate the diagnostic performance of the DPL and OSEM algorithms across different lesion sizes and BMI categories.

**Results:**

DPL produced superior PET image quality compared to OSEM across all BMI groups. The visual quality of DPL showed a slight decline with increasing BMI, while OSEM exhibited a more significant decline. DPL maintained a stable $\:{\text{S}\text{N}\text{R}}_{\text{L}\text{i}\text{v}\text{e}\text{r}}$ across BMI increases, whereas OSEM exhibited increased noise. In the DPL group, quantitative image quality for overweight patients matched that of normal patients with minimal variance from underweight patients. In contrast, OSEM demonstrated significant declines in quantitative image quality with rising BMI. DPL yielded significantly higher contrast ($\:{\text{S}\text{B}\text{R}}_{\text{L}\text{e}\text{s}\text{i}\text{o}\text{n}}\:,\:{\text{S}\text{N}\text{R}}_{\text{L}\text{e}\text{s}\text{i}\text{o}\text{n}}$, $\:{\text{C}\text{B}\text{R}}_{\text{L}\text{e}\text{s}\text{i}\text{o}\text{n}}$,$\:\:{\text{C}\text{N}\text{R}}_{\text{L}\text{e}\text{s}\text{i}\text{o}\text{n}}$) and $\:{\text{S}\text{U}\text{V}}_{\text{m}\text{a}\text{x}}^{\text{L}\text{e}\text{s}\text{i}\text{o}\text{n}}$ than OSEM for all lesions across all BMI categories.

**Conclusion:**

DPL consistently provided superior image quality and lesion diagnostic performance compared to OSEM across all BMI categories in ^18^F-FDG PET/CT scans. Therefore, we recommend using the DPL algorithm for ^18^F-FDG PET/CT image reconstruction in all BMI patients.

## Background

Positron emission tomography integrated with computed tomography (PET/CT) is a noninvasive molecular imaging technique valuable in various medical fields, including oncology, neurology, and cardiology [1–5]. This modality facilitates early tumor detection, staging, and treatment monitoring. The image quality and accuracy of PET/CT imaging are crucial for quantifying tumor metabolism, making it superior to traditional diagnostic methods [1–3]. The PET reconstruction algorithm significantly affects the standardized uptake value (SUV), reflecting the tumor’s radiotracer uptake [6–8]. The most widely used reconstruction method is the ordered subset expectation maximization (OSEM) algorithm, introduced by Hudson in 1994 [7]. While OSEM processes images quickly, its image quality may be inferior in patients with high body mass index (BMI). Post-smoothing filters are often used to mitigate noise, yet these can lower spatial resolution and potentially underestimate the SUV [9, 10]. Therefore, developing new image reconstruction algorithms to enhance image quality and diagnostic precision is essential.

Wu Z et al. [11] found that the Q.Clear algorithm produced the highest SUV values for all nodule sizes. Sui X et al. [12] found that the HYPER Iterative algorithm improved image quality and quantitative assessment in patients with a BMI of 30 or higher. However, the efficacy of these methods can be influenced by their penalty functions, necessitating adjustments based on clinical experience [13]. Recent advancements in deep learning have shown promises in enhancing PET image quality and reducing noise [13–16]. Hu Y et al. [17].demonstrated that deep-learning techniques facilitated precise tumor assessment and quantification using low-dose ^18^F-FDG PET imaging in lymphoma patients.

The deep progressive learning (DPL) algorithm, driven by artificial intelligence, has been shown to reduce noise and enhance PET contrast [18, 19]. Wang T et al. [18] reported that DPL could cut the required ^18^F-FDG activity by up to two-thirds while maintaining image quality. Xing Y et al. [20] observed that the deep-learning approach improved both the signal-to-noise ratio ($\:{\text{S}\text{N}\text{R}}_{\text{L}\text{i}\text{v}\text{e}\text{r}}$) and the lesion-to-background ratio ($\:{\text{S}\text{B}\text{R}}_{\text{L}\text{e}\text{s}\text{i}\text{o}\text{n}}$), indicating better image quality and lesion detectability compared to Gaussian filtering. Our previous research [21] demonstrated that the DPL algorithm significantly enhances PET image quality, allowing for more accurate quantification of sub-centimeter lesions in overweight patients. However, further studies are needed to assess the DPL algorithm’s effectiveness across varying BMI categories.

In this paper, PET image quality was assessed using both visual and quantitative analyses, with 466 lesions identified and classified into larger (over 1 cm) and sub-centimeter categories. The primary objective was to evaluate the performance of DPL across different BMI levels and lesion sizes, which is crucial for the early detection of malignant tumors and the timely initiation of treatment.

## Methods

### Patient selection and lesion distribution

This study was approved by the Medical Ethics Committee of Fudan University Shanghai Cancer Center (FUSCC) and adhered to its ethical standards. All patients provided written informed consent before the injection.

We enrolled 150 oncology patients (82 males and 68 females, aged 20 to 83 years) who underwent clinical ^18^F-FDG PET/CT scans at the FUSCC from March to October 2023. The scans identified 466 distinct lesions across 21 cancer types, located in various regions: head and neck (*n* = 17), chest (*n* = 84), abdomen (*n* = 36), and pelvis (*n* = 17). Patients were classified into BMI categories according to World Health Organization (WHO) guidelines [24]: underweight (BMI < 18.5), normal weight (18.5 ≤ BMI < 25.0), and overweight/obese (BMI ≥ 25.0). Baseline characteristics of the patients between the BMI groups, including age, sex, blood glucose levels, injection dose per kilogram and the uptake time between injection and imaging, showed no significant statistical variance (H-values ranging from 0.17 to 4.70, *χ*^2^ = 3.08; all *P* > 0.05). Besides, lesions were categorized by size, distinguishing between sub-centimeter lesions (maximum diameter ≤ 1 cm) and larger lesions (maximum diameter > 1 cm).The lesions were distributed as follows: the underweight group had 118 lesions (62 larger, 56 sub-centimeter), the normal group had 207 lesions (139 larger, 68 sub-centimeter) and the overweight group had 141 lesions (81 larger, 60 sub-centimeter). A summary of patients’ characteristics and lesion distribution is shown in Table 1.

All patients were referred by clinicians for ^18^F-FDG PET/CT scans based on clinical indications. Eligibility criteria included a pathological report confirming malignant tumors and adequate cooperation during the examination. Participants fasted for at least six hours to maintain blood glucose levels below 11.1 mmol/L. The radiotracer dosage was set at 3.7 MBq/kg, based on body weight to balance radiation exposure and image quality, with imaging performed approximately one-hour post-injection. During the 60-minute uptake phase, patients were instructed to drink 500 mL of water and rest quietly.

**Table 1:** Enrolled patients’ clinical characteristics and lesion distribution

Characteristics		Underweight (*n* = 42)	Normal (*n* = 66)	Overweight (*n* = 42)	*P*
Age(years)		63.4 ± 13.8	61.2 ± 10.7	63.5 (51.5,70.0)	0.23
Sex					0.22
Male		19	41	22	
Female		23	25	20	
BMI (kg/m²)		17.3 (16.6,17.7)	22.1 (20.8,23.1)	27.5 ± 2.9	<0.001
Blood glucose (mmol/L)		5.9 ± 1.1	5.7 ± 1.3	5.5 (4.8,6.5)	0.54
Injected dose (MBq)		171.1 (163.9,190.2)	223.9 (210.2,243.8)	276.9 ± 41.3	<0.001
Injected dose/weight (MBq/kg)		3.8 ± 0.3	3.8 ± 0.3	3.7 ± 0.2	0.26
Uptake time (min)		68.0 (55.1,79.3)	65.1 (58.7,75.4)	69.5 ± 16.2	0.92
Primary cancer type*					
Bone cancer		0/1	1/1	0/1	
Breast cancer		2/12	6/12	4/12^**^	
Cervical cancer		2/5	2/5	1/5	
Colorectal cancer		4/11	2/11^**^	5/11	
Duodenal cancer		0/1	1/1	0/1	
Epithelial cancer		1/3	0/3	2/3	
Esophageal cancer		6/23	13/23^**^	4/23	
Gastric cancer		6/7^**^	1/7	0/7	
Hypopharynx cancer		1/2	1/2	0/2	
Kidney cancer		0/2	2/2	0/2	
Liver cancer		1/3	1/3	1/3	
Lung cancer		11/45	18/45^**^	16/45^**^	
Lymphoma		3/17	7/17	7/17	
Malignant melanoma		1/2	0/2	1/2	
Nasopharynx cancer		1/6	3/6	2/6	
Ovarian cancer		0/3	3/3	0/3	
Pancreatic cancer		1/6	5/6^**^	0/6	
Sarcoma		1/2	1/2	0/2	
Spindle cell tumor		1/1	0/1	0/1	
Squamous cell carcinoma		0/1	1/1	0/1	
Thyroid cancer		1/1^**^	0/1	0/1	

* The fraction’s denominator represents the number of patients with the primary type of tumor, while the numerator represents the number of patients with the primary type of tumor in the corresponding different BMI groups. ** Indicates that four patients had dual cancers: one with pancreatic and lung cancer, one with esophageal and colorectal cancer, one with breast and lung cancer and one with thyroid and gastric cancer

### PET/CT acquisition and reconstruction

PET/CT imaging was performed using a digital PET/CT scanner (uMI 780, United Imaging Healthcare, Shanghai, China), which features a sensitivity of 16 kcps/MBq, a spatial resolution of 2.9 mm, a time-of-flight (TOF) resolution of 450 ps, an axial field of view (FOV) of 26.4 cm, and a scanning range from the skull base to the upper thighs. The protocol began with a TOMO scan for accurate patient positioning, followed by a low-dose diagnostic CT acquired at a fixed tube voltage of 120 kV. CT was carried out by utilizing automatic exposure control with a dynamic range of 15–100 mA to provide essential anatomical details and attenuation correction for PET images.

PET images were obtained in 3–5 bed positions (35% overlap), depending on patient height, employing a step-and-shoot mode with a 1.5-minute duration per position. Image reconstruction utilized both OSEM and DPL algorithms. OSEM reconstruction was performed with 2 iterations and 20 subsets, a 3 mm full-width at half-maximum Gaussian filter [18, 20–23], a 150 × 150 image matrix, a 600 mm FOV, a transverse pixel size of 4 mm, an axial slice thickness of 2 mm, and included TOF and point spread function (PSF) corrections. DPL was reconstructed using the same FOV, image matrix, and slice thickness parameters as OSEM, along with a smoothing factor of 3. Standard corrections for scatter, randomness, dead time, decay, attenuation, and normalization were applied during reconstruction.

### DPL reconstruction algorithm

This study employs the DPL algorithm detailed in [19], which also describes the network training and testing processes. The DPL network comprises two components: a denoising network (CNN-DE) which can remove the noise from the input image and an enhancement network (CNN-EH) which maps from a low convergent image to a high convergent image, both operating in a 2D environment. These networks were trained on a dataset of 161,040 image slice pairs including 53,680 pairs of 2.4 mm slices and 107,360 pairs of 1.2 mm slices. For testing, the dataset included 40,260 image slice pairs from 20 patients: 13,420 pairs of 2.4 mm slices and 26,840 pairs of 1.2 mm slices. The CNN-DE network utilized PET images down-sampled by 10%, while the CNN-EH network used images with incomplete iterations. Target images for both networks were full-count, complete-iteration images with dimensions of 249 × 249 × 671 pixels and a voxel size of 2.4 × 2.4 × 2.68 mm³. The DPL network utilized Kaiming initialization for parameters, with back propagation updating them via the adaptive moment estimation optimization algorithm based on a loss function. A triangular cyclic learning rate policy was implemented, where the learning rate progressively increases from a minimum of 1 × 10⁻⁵ (1e–5) to a maximum of 1 × 10⁻⁴ (1e–4) during the ascending phase, then gradually decreases back to the minimum in the descending phase. The training was conducted on a cluster of four NVIDIA Quadro RTX 6000 GPUs using Pytorch 1.5.0, initially on the uExplorer whole-body scanner, and subsequently adapted for other scanners. We utilized binary images as inputs for all networks. To ensure consistency and reliability across different scans, we applied z-score normalization to account for variations in intensity. This normalization process is essential for enabling the network to generalize effectively across diverse datasets.

### Visual image analysis

Two experienced nuclear medicine physicians, each with over five years of clinical experience, visually analyzed PET images reconstructed using both OSEM and DLP algorithms. They evaluated transverse sequence and maximum intensity projection (MIP) images in random order by using a dedicated reporting system. To eliminate bias, patient information and reconstruction algorithms remained anonymous. Image quality was assessed using a five-point Likert scale (1: poor to 5: excellent) for overall quality, sharpness, noise, and diagnostic confidence. Scores of 3 to 5 were considered clinically acceptable, while scores of 1 and 2 were deemed suboptimal for diagnosis. Higher scores reflected superior image quality based on visual assessment [24].

### Quantitative image quality analysis

A third, blinded physician conducted a quantitative analysis by selecting three homogeneous areas within the liver parenchyma, located 2 cm from the liver edge to avoid intrahepatic lesions and large blood vessels. Within each area, a circular region of interest (ROI) with a radius of 1 cm was marked. The mean SUV ($\:{\text{S}\text{U}\text{V}}_{\text{m}\text{e}\text{a}\text{n}}^{\text{L}\text{i}\text{v}\text{e}\text{r}}$), standard deviation (SD) of SUV ($\:{\text{S}\text{U}\text{V}}_{\text{S}\text{D}}^{\text{L}\text{i}\text{v}\text{e}\text{r}}$) and maximum SUV ($\:{\text{S}\text{U}\text{V}}_{\text{m}\text{a}\text{x}}^{\text{L}\text{i}\text{v}\text{e}\text{r}}$) of the ROI for both OSEM and DPL reconstructions were measured using the scanner ‘s built-in software The SUV value is calculated by multiplying the final reconstructed image by a quantification factor that accounts for patient weight and injected dose. The image uniformity index ($\:{\text{I}\text{U}\text{I}}_{\text{L}\text{i}\text{v}\text{e}\text{r}}$) and signal-to-noise ratio ($\:{\text{S}\text{N}\text{R}}_{\text{L}\text{i}\text{v}\text{e}\text{r}}$) were calculated as follows:

1 $$\:{IUI}_{\text{L}\text{i}\text{v}\text{e}\text{r}}=\frac{{SUV}_{\text{m}\text{a}\text{x}}^{\text{L}\text{i}\text{v}\text{e}\text{r}}}{{SUV}_{\text{m}\text{e}\text{a}\text{n}}^{\text{L}\text{i}\text{v}\text{e}\text{r}}}$$,

2 $$\:{SNR}_{\text{L}\text{i}\text{v}\text{e}\text{r}}=\frac{{SUV}_{\text{m}\text{e}\text{a}\text{n}}^{\text{L}\text{i}\text{v}\text{e}\text{r}}}{{SUV}_{\text{S}\text{D}}^{\text{L}\text{i}\text{v}\text{e}\text{r}}}$$.

The closer the $\:{\text{I}\text{U}\text{I}}_{\text{L}\text{i}\text{v}\text{e}\text{r}}\:$value is to 1, the better the image uniformity and quality. Conversely, a value further from 1 indicates greater image noise and lower quality. Additionally, a higher $\:{\text{S}\text{N}\text{R}}_{\text{L}\text{i}\text{v}\text{e}\text{r}}$ reflects better image quality.

### Lesion diagnostic performers analysis

All pathologically confirmed positive lesions on PET were included in the analysis. For each patient, a maximum of 10 lesions were selected. If there were more than 10 lesions in one patient, 10 target lesions (five maximum and five minimum FDG-avid lesions) were defined for further analysis. All selected lesions were identified in both DPL and OSEM reconstructions, with no discrepancies in lesion detection between the two reconstruction methods. Lesion size was measured using CT images, focusing on the largest diameter for solid tumors across multiple slices and short axis for metastatic lymph nodes and primary lymphomas. Lesions larger than 1 cm were classified as larger lesions, while those 1 cm or smaller were categorized as sub-centimeter lesions. A larger spherical volume of interest (VOI) was drawn on PET images to encompass the entire lesion while excluding high uptake areas. The SUV_max_ of the lesions ($\:{\text{S}\text{U}\text{V}}_{\text{m}\text{a}\text{x}}^{\text{L}\text{e}\text{s}\text{i}\text{o}\text{n}}$) was obtained automatically using the system built-in software. The signal-to-background ratio ($\:{\text{S}\text{B}\text{R}}_{\text{L}\text{e}\text{s}\text{i}\text{o}\text{n}}$), signal-to-noise ratio ($\:{\text{S}\text{N}\text{R}}_{\text{L}\text{e}\text{s}\text{i}\text{o}\text{n}}$), contrast-to-background ratio ($\:{\text{C}\text{B}\text{R}}_{\text{L}\text{e}\text{s}\text{i}\text{o}\text{n}}$) and contrast-to-noise ratio ($\:{\text{C}\text{N}\text{R}}_{\text{L}\text{e}\text{s}\text{i}\text{o}\text{n}}$) were calculated according to Formula 3, Formula 4, Formula 5 and Formula 6, respectively. Higher values of these parameters indicate better image quality and improved detectability of the lesions.

3 $$\:{SBR}_{Lesion}=\frac{{SUV}_{\text{m}\text{a}\text{x}}^{\text{L}\text{e}\text{s}\text{i}\text{o}\text{n}}}{{SUV}_{\text{m}\text{e}\text{a}\text{n}}^{\text{L}\text{i}\text{v}\text{e}\text{r}}}$$,

4 $$\:{SNR}_{\text{L}\text{e}\text{s}\text{i}\text{o}\text{n}}=\frac{{SUV}_{\text{m}\text{a}\text{x}}^{\text{L}\text{e}\text{s}\text{i}\text{o}\text{n}}}{{SUV}_{\text{S}\text{D}}^{\text{L}\text{i}\text{v}\text{e}\text{r}}}$$,

5 $$\:{CBR}_{\text{L}\text{e}\text{s}\text{i}\text{o}\text{n}}=\frac{{{SUV}_{\text{m}\text{a}\text{x}}^{\text{L}\text{e}\text{s}\text{i}\text{o}\text{n}}-SUV}_{\text{m}\text{e}\text{a}\text{n}}^{\text{L}\text{i}\text{v}\text{e}\text{r}}}{{SUV}_{mean}^{Liver}}$$,

6 $$\:{CNR}_{\text{L}\text{e}\text{s}\text{i}\text{o}\text{n}}=\frac{{{SUV}_{\text{m}\text{a}\text{x}}^{\text{L}\text{e}\text{s}\text{i}\text{o}\text{n}}-SUV}_{\text{m}\text{e}\text{a}\text{n}}^{\text{L}\text{i}\text{v}\text{e}\text{r}}}{{SUV}_{\text{S}\text{D}}^{\text{L}\text{i}\text{v}\text{e}\text{r}}}$$.

where$\:\:{SUV}_{\text{m}\text{a}\text{x}}^{\text{L}\text{e}\text{s}\text{i}\text{o}\text{n}}$ and $\:{SUV}_{\text{m}\text{e}\text{a}\text{n}}^{\text{L}\text{e}\text{s}\text{i}\text{o}\text{n}}$ denote the maximum and mean SUV of the lesion, while $\:{SUV}_{\text{m}\text{e}\text{a}\text{n}}^{\text{L}\text{i}\text{v}\text{e}\text{r}}$ and $\:{SUV}_{\text{S}\text{D}}^{\text{L}\text{i}\text{v}\text{e}\text{r}}$ denote the mean and standard deviation SUV of the liver.

### Statistical analysis

Statistical analysis was conducted using SPSS 27.0 (IBM SPSS Inc., Armonk, NY, USA). Data were initially assessed for normal distribution with the Shapiro-Wilk test. Quantitative data with normal distribution were expressed as mean ± SD, while non-normally distributed data were expressed as median M (Q1, Q3). Paired normally distributed data were analyzed with the paired sample t-test, and non-normally distributed data with the Wilcoxon signed-rank test. For comparisons among different BMI groups, one-way ANOVA was used for normally distributed data, while the Kruskal-Wallis H test was applied for non-normally distributed data. Lesion categories were compared using the independent-samples t-test for normal distribution and the Mann-Whitney U test for non-normal distribution. A *P*-value of less than 0.05 was deemed statistically significant.

## Results

### Visual image analysis

**Fig. 1:**
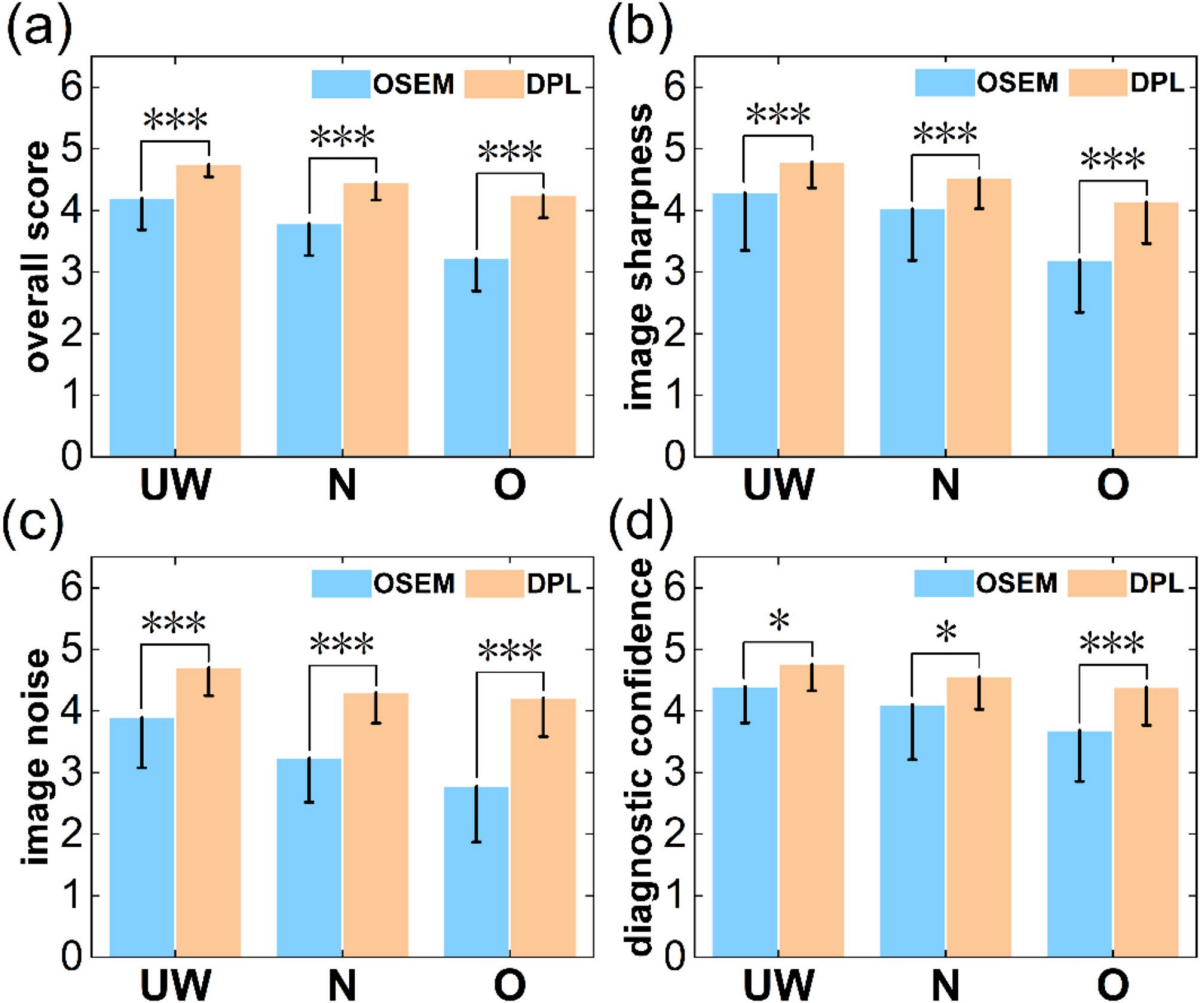
Visual image quality parameters of PET images reconstructed by using OSEM and DPL across different BMI categories. **a**–**d** represent the values of the overall score, image sharpness, image noise and diagnostic confidence, respectively, which are the four criteria for assessing PET visual image quality. Each criterion is rated on a 5-point scale, where 1 indicating poor image quality and 5 indicating excellent image quality. UW = underweight, N = normal and O = overweight. * indicates *P* < 0.05 and *** indicates *P* < 0.001

DPL outperformed OSEM in overall scores, image sharpness, image noise, and diagnostic confidence across all BMI categories (Fig. 1). In the underweight group, DPL improved by 13.23%, 11.67%, 20.73% and 8.11%, respectively (z-values: -4.91 to -2.54, all *P* < 0.05). For the normal group, the improvements were 17.71%, 12.41%, 32.71% and 11.07%, respectively (z-values: -6.92 to -3.12, all *P* < 0.05). In the overweight group, the improvements were 31.82%, 29.38%, 51.69% and 18.96%, respectively (z-values: -5.42 to -3.44, all *P* < 0.001). Both the DPL and OSEM exhibited significant declines in overall scores, image sharpness, image noise, and diagnostic confidence with increasing BMI (*P* < 0.05 for DPL and *P* < 0.001 for OSEM). In the DPL group, compared to the underweight group, the overweight group demonstrated reductions of 10.64%, 13.51%, 10.55%, and 7.84%, respectively, while the normal weight group showed decreases of 6.09%, 5.34%, 8.72%, and 4.23%, respectively. The overweight group also had reductions of 4.85%, 8.63%, 2.00%, and 3.77%, respectively compared to the normal group. Similar patterns were observed in the OSEM group, where the overweight group had reductions of 23.24%, 25.35%, 28.80%, and 16.25%, respectively compared to the underweight group, while the normal group had decreases of 9.66%, 5.96%, 16.96%, and 6.78%, respectively. Additionally, the overweight group showed reductions of 15.03%, 20.62%, 14.26%, and 10.15%, respectively compared to the normal group.

**Fig. 2:**
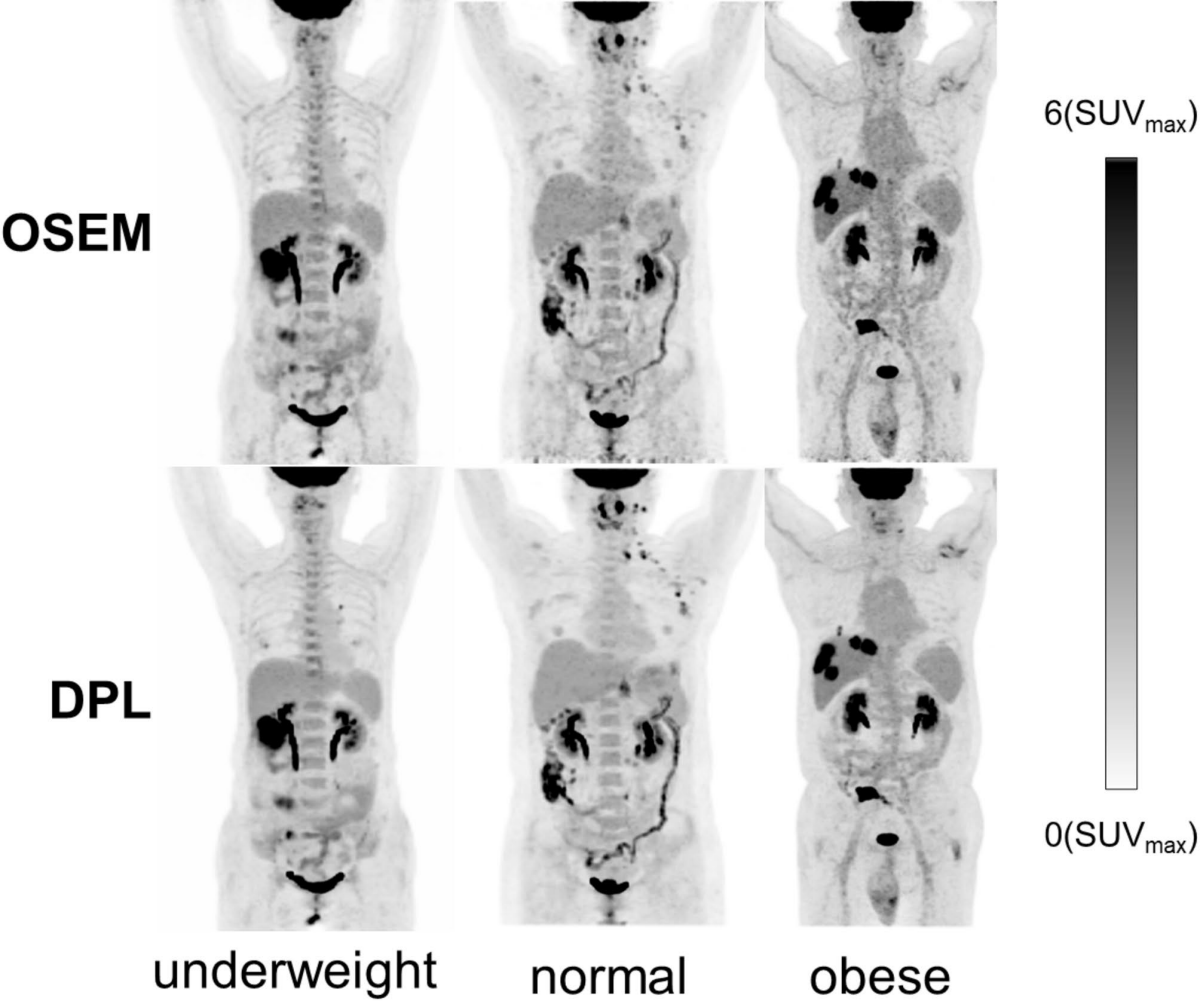
Typical PET MIP images reconstructed with OSEM and DPL across BMI categories. The DPL images exhibit significantly better visual quality compared to those reconstructed with OSEM. From left to right, the BMIs are 17.58, 23.73, and 33.25, respectively

Figure 2 compares typical PET MIP images from underweight, normal and overweight patients, revealing that DPL images consistently exhibit superior visual quality across all BMI categories compared to OSEM. Notably, while the quality of OSEM images decreases with higher BMI, DPL maintains high image quality.

### Quantitative image quality analysis

**Fig. 3:**
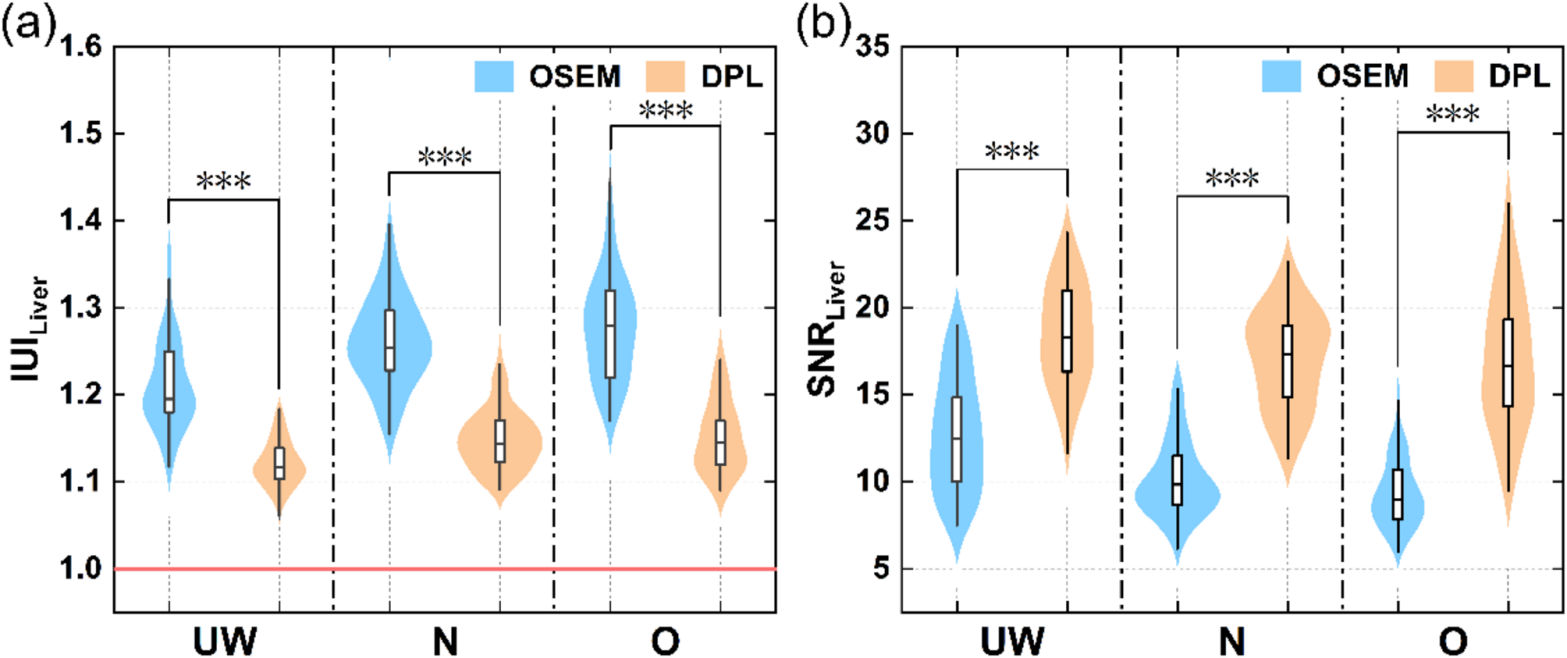
Comparison of quantitative image quality parameters ($\:{\text{I}\text{U}\text{I}}_{\text{L}\text{i}\text{v}\text{e}\text{r}}$ and $\:{\text{S}\text{N}\text{R}}_{\text{L}\text{i}\text{v}\text{e}\text{r}}$) of different PET reconstruction algorithms (OSEM and DPL) in patients with varying BMI categories. **a**–**b** indicates the value of $\:{\text{I}\text{U}\text{I}}_{\text{L}\text{i}\text{v}\text{e}\text{r}}$ and $\:{\text{S}\text{N}\text{R}}_{\text{L}\text{i}\text{v}\text{e}\text{r}}$ in underweight, normal and overweight patients, respectively. The closer the $\:{\text{I}\text{U}\text{I}}_{\text{L}\text{i}\text{v}\text{e}\text{r}}$ value is to 1, the better the uniformity of the image, indicating higher image quality. Conversely, the farther the $\:{\text{I}\text{U}\text{I}}_{\text{L}\text{i}\text{v}\text{e}\text{r}}\:$value is from 1, the greater the image noise and the poorer image quality. In addition, a higher $\:{\text{S}\text{N}\text{R}}_{\text{L}\text{i}\text{v}\text{e}\text{r}}$ indicates better image quality. UW = underweight, N = normal and O = overweight. *** indicates *P* < 0.001

DPL consistently maintained high quantitative image quality across all BMI groups, whereas OSEM reconstructions significantly degraded as BMI increased (Table 2; Fig. 3). In DPL images, $\:{\text{I}\text{U}\text{I}}_{\text{L}\text{i}\text{v}\text{e}\text{r}}$ significantly decreased compared to OSEM, while $\:{\text{S}\text{N}\text{R}}_{\text{L}\text{i}\text{v}\text{e}\text{r}}$ showed a significant increase. In the underweight group, $\:{\text{I}\text{U}\text{I}}_{\text{L}\text{i}\text{v}\text{e}\text{r}}$ decreased notably by 7.69% (z-value: -5.58, *P* < 0.001), and $\:{\text{S}\text{N}\text{R}}_{\text{L}\text{i}\text{v}\text{e}\text{r}}$ increased by 44.62% (t-value: 8.125, *P* < 0.001) relative to the OSEM group. Similar trends were observed in the normal and overweight groups, with $\:{\text{I}\text{U}\text{I}}_{\text{L}\text{i}\text{v}\text{e}\text{r}}$ decreasing by 9.18% (z-value: -7.06, *P* < 0.001) and 9.91% (z-value: -5.65, *P* < 0.001), respectively, while $\:{\text{S}\text{N}\text{R}}_{\text{L}\text{i}\text{v}\text{e}\text{r}}$ increasing by 65.36% (z-value: -7.02, *P* < 0.001) and 80.11% (z-value: -5.65, *P* < 0.001), respectively. In addition, there were significant differences in $\:{\text{S}\text{N}\text{R}}_{\text{L}\text{i}\text{v}\text{e}\text{r}}$ across BMI categories in the OSEM group (*P* < 0.001), whereas DPL showed no significant difference (*P* = 0.08). Both reconstruction methods displayed significant differences in $\:{\text{I}\text{U}\text{I}}_{\text{L}\text{i}\text{v}\text{e}\text{r}}$ across BMI categories (OSEM: *P* < 0.001; DPL: *P* < 0.001), but there was no significant difference between the normal and the overweight group for the DPL reconstruction. For DPL reconstructions, $\:{\text{I}\text{U}\text{I}}_{\text{L}\text{i}\text{v}\text{e}\text{r}}$ was increased by only 0.28% and $\:{\text{S}\text{N}\text{R}}_{\text{L}\text{i}\text{v}\text{e}\text{r}}$ remained stable for the overweight group compared to the normal group, while for the normal group compared to the underweight group, $\:{\text{I}\text{U}\text{I}}_{\text{L}\text{i}\text{v}\text{e}\text{r}}$ increased by 2.91% and $\:{\text{S}\text{N}\text{R}}_{\text{L}\text{i}\text{v}\text{e}\text{r}}$decreased by 7.48%. For OSEM reconstructions, $\:{\text{I}\text{U}\text{I}}_{\text{L}\text{i}\text{v}\text{e}\text{r}}$ was increased by 1.09% and $\:{\text{S}\text{N}\text{R}}_{\text{L}\text{i}\text{v}\text{e}\text{r}}$was decreased by 8.2% for the overweight group compared to the normal group, while $\:{\text{I}\text{U}\text{I}}_{\text{L}\text{i}\text{v}\text{e}\text{r}}$ was increased by 4.30% and $\:{\text{S}\text{N}\text{R}}_{\text{L}\text{i}\text{v}\text{e}\text{r}}$as decreased by 19.08% for the normal group compared to the underweight group.

**Table 2:** Quantitative image quality parameters ($\:{\text{I}\text{U}\text{I}}_{\text{L}\text{i}\text{v}\text{e}\text{r}}$ and $\:{\text{S}\text{N}\text{R}}_{\text{L}\text{i}\text{v}\text{e}\text{r}}$) comparing OSEM and DPL reconstructions across all BMI categories

Quantitative parameters	Underweight		Normal		Overweight	
	OSEM	DPL	OSEM	DPL	OSEM	DPL
$\:{\mathbf{I}\mathbf{U}\mathbf{I}}_{\mathbf{L}\mathbf{i}\mathbf{v}\mathbf{e}\mathbf{r}}$	1.20 (1.18, 1.25)	1.12 ± 0.03	1.25 (1.23, 1.30)	1.14 (1.12, 1.17)	1.28 ± 0.06	1.15 (1.12, 1.18)
$\:{\mathbf{S}\mathbf{N}\mathbf{R}}_{\mathbf{L}\mathbf{i}\mathbf{v}\mathbf{e}\mathbf{r}}$	12.75 ± 3.32	18.44 ± 3.08	9.83 (8.67, 11.58)	17.06 ± 2.86	8.94 (7.81, 10.79)	17.06 ± 4.07

### Lesion diagnostic performances

**Fig. 4:**
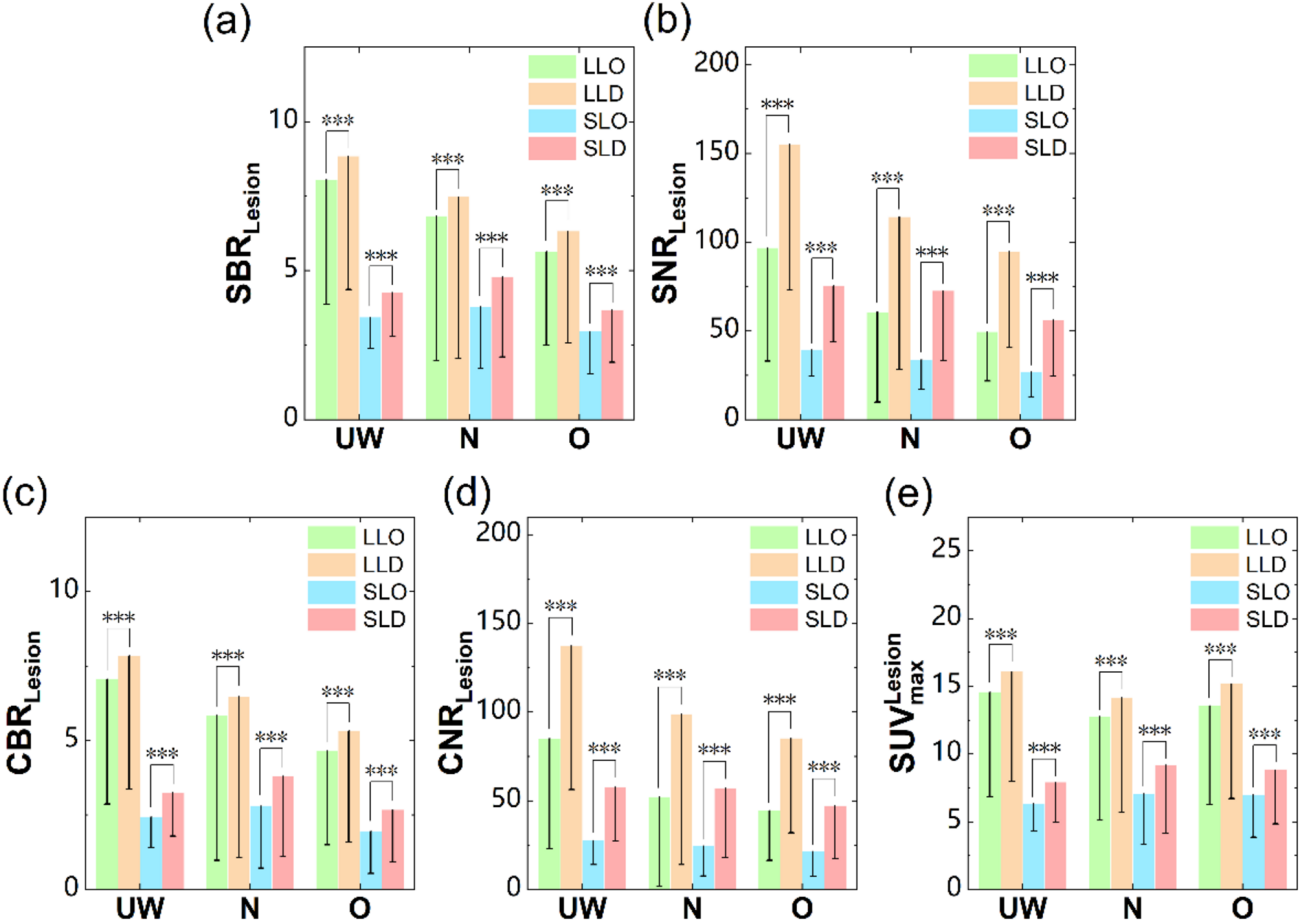
Diagnostics performances of OSEM and DPL for larger and sub-centimeter lesions in patients with different BMIs. a–e represents the values of $\:\:{\text{S}\text{B}\text{R}}_{\text{L}\text{e}\text{s}\text{i}\text{o}\text{n}}$, $\:{\text{S}\text{N}\text{R}}_{\text{L}\text{e}\text{s}\text{i}\text{o}\text{n}}$, $\:{\text{C}\text{B}\text{R}}_{\text{L}\text{e}\text{s}\text{i}\text{o}\text{n}}$,$\:\:{\text{C}\text{N}\text{R}}_{\text{L}\text{e}\text{s}\text{i}\text{o}\text{n}}$ and $\:{\text{S}\text{U}\text{V}}_{\text{m}\text{a}\text{x}}^{\text{L}\text{e}\text{s}\text{i}\text{o}\text{n}}$, respectively. UW = underweight, N = normal, O = overweight, LLO = Larger lesions with OSEM, LLD = Larger lesions with DPL, SLO = sub-centimeter lesions with OSEM, SLD = sub-centimeter lesions with DPL. *** indicates *P* < 0.001

The values of $\:{\text{S}\text{B}\text{R}}_{\text{L}\text{e}\text{s}\text{i}\text{o}\text{n}}$, $\:{\text{S}\text{N}\text{R}}_{\text{L}\text{e}\text{s}\text{i}\text{o}\text{n}}$, $\:{\text{C}\text{B}\text{R}}_{\text{L}\text{e}\text{s}\text{i}\text{o}\text{n}}$,$\:\:{\text{C}\text{N}\text{R}}_{\text{L}\text{e}\text{s}\text{i}\text{o}\text{n}}$ and $\:{\text{S}\text{U}\text{V}}_{\text{m}\text{a}\text{x}}^{\text{L}\text{e}\text{s}\text{i}\text{o}\text{n}}$ across BMI and lesion sizes, obtained using OSEM and DPL reconstructions, are presented in Table 3. The DPL algorithm consistently yielded significantly higher values for $\:{\text{S}\text{B}\text{R}}_{\text{L}\text{e}\text{s}\text{i}\text{o}\text{n}}$, $\:{\text{S}\text{N}\text{R}}_{\text{L}\text{e}\text{s}\text{i}\text{o}\text{n}}$, $\:{\text{C}\text{B}\text{R}}_{\text{L}\text{e}\text{s}\text{i}\text{o}\text{n}}$,$\:\:{\text{C}\text{N}\text{R}}_{\text{L}\text{e}\text{s}\text{i}\text{o}\text{n}}$ and $\:{\text{S}\text{U}\text{V}}_{\text{m}\text{a}\text{x}}^{\text{L}\text{e}\text{s}\text{i}\text{o}\text{n}}$ compared to OSEM across all lesion sizes and BMI groups (z-values: -10.13 to -5.74, *P* < 0.001). The diagnostic performance of OSEM and DPL for both large and sub-centimeter lesions across different BMI categories is presented in Fig. 4 (Fig. 4).

**Table 3:** Lesion diagnostic performances parameters of different reconstruction and different BMI (*n* = 466)

BMI categories	Parameters	Larger lesions (OSEM) (*n* = 282)	Larger lesions (DPL) (*n* = 282)	Sub-centimeter lesions (OSEM) (*n* = 184)	Sub-centimeter lesions (DPL) (*n* = 184)	
Underweight (*n* = 118)	$\:{\text{S}\text{U}\text{V}}_{\text{m}\text{a}\text{x}}^{\text{L}\text{e}\text{s}\text{i}\text{o}\text{n}}$	12.2(9.9,16.7)	14.4(10.8,18.4)	6.1(4.9,7.6)	7.6(5.9,9.2)	
	$\:{\text{S}\text{B}\text{R}}_{\text{L}\text{e}\text{s}\text{i}\text{o}\text{n}}$	7.0(5.5,10.3)	7.6(6.2,10.9)	3.2(2.6,4.4)	4.1(3.2,5.4)	
	$\:{\text{S}\text{N}\text{R}}_{\text{L}\text{e}\text{s}\text{i}\text{o}\text{n}}$	79.4(54.7,118.5)	133.0(102.3,183.5)	36.9(30.2,43.3)	67.0(55.3,91.8)	
	$\:{\text{C}\text{B}\text{R}}_{\text{L}\text{e}\text{s}\text{i}\text{o}\text{n}}$	6.0(4.5,9.3)	6.6(5.2,9.9)	2.2(1.6,3.4)	3.1(2.2,4.4)	
	$\:{\text{C}\text{N}\text{R}}_{\text{L}\text{e}\text{s}\text{i}\text{o}\text{n}}$	68.3(43.3,106.9)	118.1(85.5,164.9)	25.4(18.6,31.0)	49.0(37.8,71.5)	
Normal (*n* = 207)	$\:{\text{S}\text{U}\text{V}}_{\text{m}\text{a}\text{x}}^{\text{L}\text{e}\text{s}\text{i}\text{o}\text{n}}$	11.2(7.9,15.3)	12.1(8.8,17.1)	5.9(4.6,7.7)	7.5(6.0,9.9)	
	$\:{\text{S}\text{B}\text{R}}_{\text{L}\text{e}\text{s}\text{i}\text{o}\text{n}}$	6.1(4.1,8.2)	6.3(4.3,8.9)	3.0(2.4,4.5)	4.0(3.0,5.5)	
	$\:{\text{S}\text{N}\text{R}}_{\text{L}\text{e}\text{s}\text{i}\text{o}\text{n}}$	46.5(33.4,71.6)	88.5(63.0,141.0)	28.7(22.4,41.5)	60.5(46.5,91.0)	
	$\:{\text{C}\text{B}\text{R}}_{\text{L}\text{e}\text{s}\text{i}\text{o}\text{n}}$	5.1(3.1,7.2)	5.3(3.3,7.9)	2.0(1.4,3.5)	3.0(2.0,4.5)	
	$\:{\text{C}\text{N}\text{R}}_{\text{L}\text{e}\text{s}\text{i}\text{o}\text{n}}$	37.8(24.5,63.6)	72.8(48.0,121.3)	18.7(13.8,32.8)	43.5(30.8,73.8)	
Overweight (*n* = 141)	$\:{\text{S}\text{U}\text{V}}_{\text{m}\text{a}\text{x}}^{\text{L}\text{e}\text{s}\text{i}\text{o}\text{n}}$	12.0(8.2,17.3)	12.9(9.3,20.5)	6.0(4.8,8.5)	7.8(6.0,10.8)	
	$\:{\text{S}\text{B}\text{R}}_{\text{L}\text{e}\text{s}\text{i}\text{o}\text{n}}$	5.0(3.1,7.1)	5.2(3.4,8.0)	2.4(2.0,3.7)	3.1(2.5,4.6)	
	$\:{\text{S}\text{N}\text{R}}_{\text{L}\text{e}\text{s}\text{i}\text{o}\text{n}}$	43.1(27.5,61.2)	84.8(49.5,123.7)	22.0(17.9,33.7)	47.1(37.3,69.6)	
	$\:{\text{C}\text{B}\text{R}}_{\text{L}\text{e}\text{s}\text{i}\text{o}\text{n}}$	4.0(2.1,6.1)	4.2(2.4,7.0)	1.4(1.0,2.7)	2.2(1.5,3.6)	
	$\:{\text{C}\text{N}\text{R}}_{\text{L}\text{e}\text{s}\text{i}\text{o}\text{n}}$	37.1(24.0,59.4)	74.8(44.2,109.9)	17.1(11.4,27.9)	39.4(28.6,63.9)	

### Representative clinical cases

**Fig. 5:**
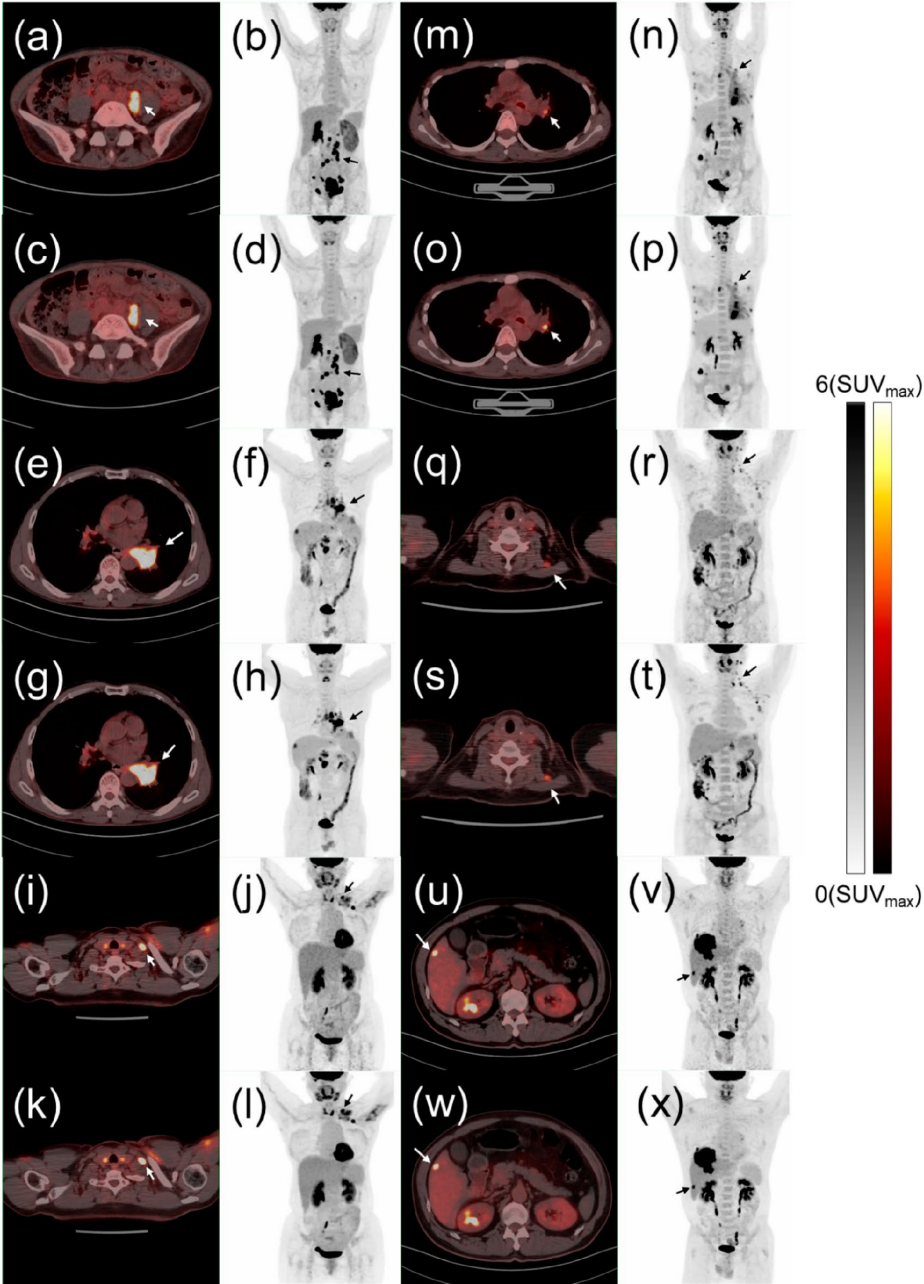
Representative PET/CT images of patients in the different BMI groups with large and sub-centimeter lesions, reconstructed with OSEM and DPL. **a**-**d**: PET/CT images of an underweight female patient with cervical cancer (BMI: 16.30) and larger lesions. The arrow indicates a metastatic lymph node in the retroperitoneum with a maximum diameter of 2.63 cm. m-p: PET/CT images of an underweight female patient with lung cancer (BMI:16.18) and sub-centimeter lesions. The arrow indicates a metastatic lymph node in the left hilum with a maximum diameter of 0.58 cm. **e**-**h**: PET/CT images of a normal-weight male patient with lung cancer (BMI: 21.23) and larger lesions. The arrow indicates a mass in the left lower lobe with a maximum diameter of 3.88 cm. **q**-**t**: PET/CT images of a normal-weight female patient with lymphoma (BMI: 23.73) and sub-centimeter lesions. The arrow indicates a lymph node in the left neck with a maximum diameter of 0.51 cm. **i**-**l**: PET/CT images of an overweight female patient with malignant melanoma (BMI: 25.39) and larger lesions. The arrow indicates a metastatic lymph node in the left subclavian region with a maximum diameter of 1.62 cm. **u**-**x**: PET/CT images of an overweight male patient with liver cancer (BMI: 28.23) and small lesions. The arrow indicates a metastatic lesion with a maximum diameter of 0.88 cm. Columns 1 and 3 display the fused PET/CT images of patients in the transverse plane, while columns 2 and 4 present the MIPs of the patients’ PET scans. Rows 1, 3 and 5 are the OSEM, Rows 2, 4 and 6 are DPL

Representative PET/CT images of patients in the different BMI groups with large and sub-centimeter lesions, reconstructed with OSEM and DPL, are presented in Fig. 5. For instance, in Fig. 5a-d, a female underweight patient with cervical cancer has a larger lesion, with $\:{\text{S}\text{U}\text{V}}_{\text{m}\text{a}\text{x}}^{\text{L}\text{e}\text{s}\text{i}\text{o}\text{n}}$ values of 11.2 for OSEM and 17.6 for DPL. Figure 5e-h, a normal-weight male lung cancer patient has a larger lesion with $\:{\text{S}\text{U}\text{V}}_{\text{m}\text{a}\text{x}}^{\text{L}\text{e}\text{s}\text{i}\text{o}\text{n}}$ values of 13.7 for OSEM and 16.9 for DPL. Figure 5i-l, an overweight female with malignant melanoma has a larger lesion, with $\:{\text{S}\text{U}\text{V}}_{\text{m}\text{a}\text{x}}^{\text{L}\text{e}\text{s}\text{i}\text{o}\text{n}}$ values of 12.4 for OSEM and 16.8 for DPL. In Fig. 5m-p, a female underweight patient with lung cancer shows a sub-centimeter lymph node metastasis, with $\:{\text{S}\text{U}\text{V}}_{\text{m}\text{a}\text{x}}^{\text{L}\text{e}\text{s}\text{i}\text{o}\text{n}}$ values of 3.9 for OSEM and 6.3 for DPL. Figure 5q-t depict a normal-weight female patient with a sub-centimeter lymph node metastasis, showing $\:{\text{S}\text{U}\text{V}}_{\text{m}\text{a}\text{x}}^{\text{L}\text{e}\text{s}\text{i}\text{o}\text{n}}$ values of 2.9 for OSEM and 3.8 for DPL. Finally, Fig. 5u-x illustrate an overweight male liver cancer patient with a sub-centimeter lesion, showing $\:{\text{S}\text{U}\text{V}}_{\text{m}\text{a}\text{x}}^{\text{L}\text{e}\text{s}\text{i}\text{o}\text{n}}$ values of 8.1 for OSEM and 9.8 for DPL.

## Discussion

In this study, we compared PET scans reconstructed using OSEM and DPL through visual and quantitative analysis of patients in different BMI categories (underweight, normal and overweight). DPL significantly improved visual image quality across all BMI categories compared to OSEM, particularly in the overweight group.

Previous studies consistently indicate that higher BMI correlates with reduced image quality in OSEM reconstructions [25, 26]. Our study supports this finding, showing a significant decline in OSEM image quality with increasing BMI. The overweight group experienced significant reductions in overall image score, image sharpness, noise suppression and diagnostic confidence compared to the normal group. In contrast, the normal group showed reductions compared to the underweight group.

However, DPL imaging was less sensitive to BMI changes. DPL consistently offers superior quantitative image quality compared to OSEM, particularly as BMI increases, and demonstrates minimal or insignificant variation in quantitative image quality across BMI groups, while OSEM shows a notable decline with increasing BMI. The overweight group had minor image quality decreases compared to the normal group. Similarly, the normal group exhibited slight declines relative to the underweight group. This confirms that while OSEM image quality significantly declines with increasing BMI, DPL remains relatively stable. PET images reconstructed by DPL exhibited superior noise reduction and image quality enhancement compared to those reconstructed by OSEM across all BMI categories.

Quantitative analysis corroborated the visual findings. Significant improvements in $\:{\text{I}\text{U}\text{I}}_{\text{L}\text{i}\text{v}\text{e}\text{r}}$ were observed with DPL across various BMI categories, indicating superior uniformity compared to OSEM. Minimal variations in $\:{\text{I}\text{U}\text{I}}_{\text{L}\text{i}\text{v}\text{e}\text{r}}$ and $\:{\text{S}\text{N}\text{R}}_{\text{L}\text{i}\text{v}\text{e}\text{r}}$ across BMI categories suggest that DPL ensures comparable image quality in overweight patients to that of normal patients, with slight deviations noted in the underweight group.

Furthermore, both OSEM and DPL reconstructions demonstrated excellent quantitative image quality in the underweight group. However, as BMI increased, the image quality of OSEM reconstructions significantly declined, consistent with previous studies [26], whereas DPL maintained stable image quality. The $\:{\text{S}\text{N}\text{R}}_{\text{L}\text{i}\text{v}\text{e}\text{r}}$ values further supported these findings; OSEM values gradually decreased with increasing BMI, whereas DPL maintained consistent $\:{\text{S}\text{N}\text{R}}_{\text{L}\text{i}\text{v}\text{e}\text{r}}$ values across BMI categories, indicating its capability to sustain adequate image quality. Previous study [27] suggest that an $\:{\text{S}\text{N}\text{R}}_{\text{L}\text{i}\text{v}\text{e}\text{r}}$ above 14.0 is necessary for good image quality. Our study demonstrated that DPL achieved this threshold across all BMI categories with a 1.5-minute bed acquisition time. In contrast, OSEM failed to meet this standard for all BMI groups, often requiring up to 15 min acquisition time for overweight patients [1]. Thus, DPL can shorten the PET acquisition time for patients, thereby enhancing their comfort while maintaining image quality. Additionally, DPL could reduce the injection dose of ^18^F-FDG for patients while preserving image quality. Wang et al. [18] estimated that DPL could potentially reduce the administered ^18^F-FDG activity by two-thirds in clinical practice, thereby lowering radiation exposure and enhancing patient comfort.

Accurate lesion diagnosis is essential for tumor staging, treatment planning, and monitoring treatment response. However, the partial volume effect often obscures sub-centimeter lesions in the background, reducing diagnostic performance. Thus, enhancing the contrast and $\:{\text{S}\text{U}\text{V}}_{\text{m}\text{a}\text{x}}^{\text{L}\text{e}\text{s}\text{i}\text{o}\text{n}}$ of sub-centimeter lesions is crucial. Prior studies show that sensitivity decreases using OSEM reconstruction for sub-centimeter lesions, leading to potential false negatives [1, 28]. Enhancing $\:{\text{S}\text{U}\text{V}}_{\text{m}\text{a}\text{x}}^{\text{L}\text{e}\text{s}\text{i}\text{o}\text{n}}$ and lesions contrast has become a key metric for assessing the effectiveness of reconstruction methods. Our prior study confirmed that DPL improved $\:{\text{S}\text{U}\text{V}}_{\text{m}\text{a}\text{x}}^{\text{L}\text{e}\text{s}\text{i}\text{o}\text{n}}$ in sub-centimeter lesions among overweight patients. This paper further validates this finding with a larger sample size. Importantly, we found that the DPL reconstruction algorithm consistently and significantly enhanced contrast parameter values and $\:{\text{S}\text{U}\text{V}}_{\text{m}\text{a}\text{x}}^{\text{L}\text{e}\text{s}\text{i}\text{o}\text{n}}$ for all lesions across all BMI groups compared to OSEM. This study demonstrates that, compared to OSEM, the improvement rate of $\:{\text{S}\text{U}\text{V}}_{\text{m}\text{a}\text{x}}^{\text{L}\text{e}\text{s}\text{i}\text{o}\text{n}}$ for sub-centimeter lesions using the DPL algorithm is highest in the normal group, followed by the overweight group, and lowest in the underweight group. Notably, the diagnostic performance improvement rate for sub-centimeter lesions was higher in normal weight patients than in overweight patients, indicating greater benefits for normal weight patients from DPL.

Besides enhancing the contrast and $\:{\text{S}\text{U}\text{V}}_{\text{m}\text{a}\text{x}}^{\text{L}\text{e}\text{s}\text{i}\text{o}\text{n}}$ values of sub-centimeter lesions, DPL significantly improved the contrast and $\:{\text{S}\text{U}\text{V}}_{\text{m}\text{a}\text{x}}^{\text{L}\text{e}\text{s}\text{i}\text{o}\text{n}}$ of larger lesions. The greatest $\:{\text{S}\text{U}\text{V}}_{\text{m}\text{a}\text{x}}^{\text{L}\text{e}\text{s}\text{i}\text{o}\text{n}}$ enhancements for larger lesions were observed in the overweight group, followed by the normal group, and the underweight group.

Typical cases of PET image reconstruction with OSEM and DPL are presented in Fig. 5. These cases demonstrate that PET images reconstructed by DPL exhibit superior noise reduction and lesion contrast enhancement compared to those reconstructed by OSEM. Additionally, DPL consistently showed superior image quality and higher lesion contrast and $\:{SUV}_{max}^{Lesion}$ values compared to OSEM, regardless of patient BMI or lesion size.

There are still some limitations in this study. First, in the overweight group, we included more patients with a BMI < 30 compared to those with a BMI > 30, which may affect our results. However, our previous research has shown that, compared to the overweight group, the obese group exhibited improvements in SBR, SNR, CNR, and SUV_max_ in the DPL average values [21]. Therefore, if more individuals with a BMI > 30 were included in our study, the superiority of the DPL algorithm over the OSEM algorithm would likely be more pronounced. Additionally, body weight-based SUV values may be influenced by body weight. Sarikaya et al.‘s [22] study found that SUV values of lesions and normal tissues are higher in obese individuals. Similarly, in our study, we observed an upward trend in $\:{SUV}_{max}^{Liver}$ and $\:{SUV}_{mean}^{Liver}$ with increasing patient BMI. To mitigate this effect, we used the ratio of comparative data rather than direct comparison values in this study, aiming to minimize this impact. Our study did not find an increase in lesion $\:{SUV}_{max}^{Lesion}$ with increasing BMI, which may be due to the different types of lesions in the various BMI groups. Furthermore, our study was conducted with a data acquisition time of 1.5 min/bed. As shown in our previous research [21], extending the acquisition time to 2.5 min/bed reduces the differences in CNR and SNR between OSEM and DPL. Consequently, our results may not be reproducible with an acquisition time exceeding 2.5 min/bed. Moreover, we have acknowledged that ringing artifacts, inherent to point-spread-function (PSF)-based reconstructions, may still manifest in DPL due to its integration of TOF-PSF-OSEM as the base reconstruction step. Finally, the DPL algorithm is currently only applicable to the reconstruction of ^18^F-FDG PET images. Other images of PET radiopharmaceuticals require new specialized datasets for retraining, which significantly limits its applicability. ^18^F is less sensitive to reductions in injection dose compared to ^68^Ga, making the development of a DPL algorithm for ^68^Ga even more important.

## Conclusion

This study evaluates the image quality and lesion diagnostic performance in PET scans reconstructed by using OSEM and DPL across various BMI categories. DPL outperformed OSEM in terms of image quality and lesion diagnostic performance, maintaining stable performance with increasing BMI, while OSEM’s performance declined significantly. This indicates that DPL is less affected by increasing BMI compared to OSEM. DPL significantly improved the lesion contrast and $\:{\text{S}\text{U}\text{V}}_{\text{m}\text{a}\text{x}}^{\text{L}\text{e}\text{s}\text{i}\text{o}\text{n}}$ values across all BMI groups, with more pronounced improvement for sub-centimeter lesions compared to larger lesions. Based on these findings, we recommend DPL reconstruction for ^18^F-FDG PET imaging, particularly for patients requiring enhanced image quality and accurate lesion diagnosis.

## Data Availability

No datasets were generated or analysed during the current study.

## Abbreviations

PET/CT: Positron emission tomography integrated with computed tomography
18F-FDG: 2-deoxy-2-[fluorine-18]fluoro-D-glucose
DPL: Deep progressive learning
OSEM: Ordered subset expectation maximization
$\:{\text{I}\text{U}\text{I}}_{\text{L}\text{i}\text{v}\text{e}\text{r}}\:$: Liver image-uniformity-index
$\:{\text{S}\text{N}\text{R}}_{\text{L}\text{i}\text{v}\text{e}\text{r}}\:\:$: Liver signal-to-noise ratio
$\:{\rm{SUV}}_{\rm{max}}^{\rm{Lesion}}\:$: Lesion maximum standard uptake value
$\:{\text{S}\text{B}\text{R}}_{\text{L}\text{e}\text{s}\text{i}\text{o}\text{n}}\:$: Lesion signal-to-background ratio
$\:{\text{S}\text{N}\text{R}}_{\text{L}\text{e}\text{s}\text{i}\text{o}\text{n}}\:$: Lesion signal-to-noise ratio
$\:{\text{C}\text{B}\text{R}}_{\text{L}\text{e}\text{s}\text{i}\text{o}\text{n}}\:$: Lesion contrast-to-background ratio
$\:{\text{C}\text{N}\text{R}}_{\text{L}\text{e}\text{s}\text{i}\text{o}\text{n}}\:$: Lesion contrast-to-noise ratio
BMI: Body mass index
SUV: Standardized uptake value
FUSCC: Fudan University Shanghai Cancer Center
WHO: World Health Organization
TOF: Time-of-flight
PSF: Point spread function
FOV: Field of view
SD: Standard deviation
CNN: Convolutional neural network
CNN-DE: CNN denoising network
CNN-EH: CNN enhancement network
GAN: Generative adversarial network
MIP: Maximum intensity projection
ROI: Region of interest
VOI: Volume of interest
$\:{\text{S}\text{U}\text{V}}_{\text{m}\text{e}\text{a}\text{n}}^{\text{L}\text{i}\text{v}\text{e}\text{r}}\:$: Mean SUV value of liver
$\:{\text{S}\text{U}\text{V}}_{\text{S}\text{D}}^{\text{L}\text{i}\text{v}\text{e}\text{r}}\:$: Standard deviation SUV value of liver
$\:{\text{S}\text{U}\text{V}}_{\text{m}\text{a}\text{x}}^{\text{L}\text{i}\text{v}\text{e}\text{r}}\:$: Maximum SUV value of liver
ANOVA: Analysis of Variance
UV: Underweight
N: Normal
O: Overweight
LLO: Larger lesions with OSEM
LLO: LLD Larger lesions with DPL
SLO: Sub-centimeter lesions with OSEM
SLD: Sub-centimeter lesions with DPL
